# Factors associated with COVID-19 vaccine receipt among mobile phone users in Malawi: Findings from a national mobile-based syndromic surveillance survey, July 2021-April 2022

**DOI:** 10.1371/journal.pgph.0002722

**Published:** 2024-01-11

**Authors:** Lucky Makonokaya, Lester Kapanda, Thulani Maphosa, Louiser Upile Kalitera, Rhoderick Machekano, Harrid Nkhoma, Rachel Chamanga, Suzgo B. Zimba, Annie Chauma Mwale, Alice Maida, Godfrey Woelk

**Affiliations:** 1 Elizabeth Glaser Pediatric AIDS Foundation, Lilongwe, Malawi; 2 Elizabeth Glaser Pediatric AIDS Foundation, Washington, DC, United States of America; 3 Public Health Institute, Ministry of Health, Lilongwe, Malawi; 4 Centers for Disease Control and Prevention, Lilongwe, Malawi; Universidade de São Paulo: Universidade de Sao Paulo, BRAZIL

## Abstract

Malawi recommended COVID-19 vaccines for adults aged ≥18 years in March 2021. We assessed factors associated with receiving COVID-19 vaccines in Malawi as part of a telephone-based syndromic surveillance survey. We conducted telephone-based syndromic surveillance surveys with questions on COVID-19 vaccine receipt among adults (≥18 years old) upon verbal consent from July 2021 to April 2022. We used random digit dialing to select mobile phone numbers and employed electronic data collection forms on secure tablets. Survey questions included whether the respondent had received at least one dose of a COVID-19 vaccine. We used multivariable analysis to identify factors associated with COVID-19 vaccine receipt. Of the 51,577 participants enrolled; 65.7% were male. Males were less likely to receive the COVID-19 vaccine than females (AOR 0.83, 95% CI 0.80–0.86). Compared to those aged 18–24 years, older age had increased odds of vaccine receipt: 25–34 years (AOR 1.32, 95% CI 1.24–1.40), 35–44 years (AOR 2.00, 95% CI 1.88–2.13), 45–54 years (AOR 3.02, 95% CI 2.82–3.24), 55–64 years (AOR 3.24, 95% CI 2.93–3.57) and 65 years+ (AOR 3.98, 95% CI 3.52–4.49). Respondents without formal education were less likely to receive vaccination compared to those with primary (AOR 1.30, 95% CI 1.14–1.48), secondary (AOR 1.76, 95% CI 1.55–2.01), and tertiary (AOR 3.37, 95% CI 2.95–3.86) education. Respondents who thought COVID-19 vaccines were unsafe were less likely to receive vaccination than those who thought it was very safe (AOR 0.26, 95% CI 0.25–0.28). Residents of the Central and Southern regions had reduced odds of vaccine receipt compared to those in the North (AORs 0.79, (95% CI 0.74–0.84) and 0.55, (95% CI 0.52–0.58) respectively). Radio (72.6%), health facilities (52.1%), and social media (16.0%) were the more common self-reported sources of COVID-19 vaccine information. COVID-19 vaccine receipt is associated with gender, age, education, and residence. It is important to consider these factors when implementing COVID-19 vaccination programs.

## Introduction

Severe acute respiratory syndrome coronavirus 2 (SARS-CoV-2) infection, which is associated with coronavirus disease 2019 (COVID-19), resulted in a pandemic with a significant global disease burden, with over 530 million registered cases and 6.3 million deaths recorded globally [1]. The pandemic has had an adverse impact on health systems, and its effects have been most remarkable in low- and middle-income countries (LMICs), where there were prior existing inadequate health systems resources [2, 3]. While infection prevention and control measures such as mask-wearing in public places, hand washing, and sanitizing help reduce the spread of the infection and resultant disease, implementation of these measures remains a challenge globally, especially in LMICs [4–6].

The World Health Organization (WHO) recommended using COVID-19 vaccines to help reduce the risk of severe COVID-19 disease and death in individuals who develop SARS-CoV-2 infection and potentially reduce the risk of SARS-CoV-2 infection [7] and propel herd immunity [8]. COVID-19 vaccines minimize morbidity and mortality due to COVID-19 disease and are a critical component in the fight against the pandemic [9–11].

Recent studies have shown that up to 80% of people in LMICs, including adults in African countries, were willing to receive COVID-19 vaccines [11, 12]. However, there has been a slower uptake of vaccines in most of these countries compared to high-income countries [1]. Reasons for the slower uptake include a lack of vaccine availability and implementation logistics, as well as population concerns about vaccine efficacy, fear of side effects, and distrust of the government [13, 14]. Malawi, as a low-income country [15] in South Central Africa, has been particularly challenged by the SARS-COV2 pandemic.

With an estimated population of 17.5 million, more than half aged below 18 years [16], the country had registered a cumulative 85 991 COVID-19 cases (426 per 100,000 population) and 2 641 deaths (13 per 100,000 population) by June 1, 2022 [1]. However, like many other LMICs, Malawi has struggled to test all suspected cases for SARS-CoV-2 infection due to low laboratory diagnostic capacity, and studies suggest the disease prevalence could be much higher than that indicated by the number of reported cases [17, 18]. In March 2021, the Malawi Ministry of Health (MOH) launched a vaccination campaign, offering free AZD1222 and Ad26.COV.S COVID-19 vaccines to all adult residents (≥18 years). The campaign initially prioritized those aged at least 60 years and frontline workers (healthcare workers, police, and prison service officers) based on the availability of vaccines. However, it was opened to all adults once more vaccines were available in May 2021. The MOH recommends COVID-19 vaccines to all residents aged 12 years and older [19]. The nation planned to vaccinate at least 60% of its population by the end of 2022. As of May 22, 2022, only about 7.8% of the Malawi population had received at least one dose of COVID-19 vaccine [20]. With this low achievement and a target vaccination status unlikely to be reached, it was critical to examine factors associated with vaccine receipt among adults. While studies on predictors of vaccine acceptance and willingness to get vaccinated in the region have shown relatively higher proportions of vaccine acceptance [21, 22], it is unclear whether this is the case in Malawi. Knowledge of factors affecting vaccine receipt could be used to inform educational and programming efforts to improve vaccination uptake.

As part of a survey developed as a low-cost surveillance strategy to monitor the COVID-19 pandemic through information about COVID-19-like/influenza-like symptoms in the preceding two weeks, COVID-19 testing, and household deaths, we obtained data on views about COVID-19 vaccination, intent to vaccinate and vaccine receipt. In this paper, we describe factors associated with receipt of COVID-19 vaccination.

## Methods

### Study design

We conducted a mobile phone-based cross-sectional COVID-19 syndromic surveillance survey in Malawi [23]. We included data on COVID-19 vaccination among adults living in Malawi with access to an active mobile phone number from July 2021 to April 2022. Survey questions included age, gender, education, region of residence, getting vaccination if recommended, concerns about severe reactions to the vaccine, family decision-making on the vaccination, influence of close friends and family, trust in the vaccine, the safety of the vaccine, vaccination, number of doses received, and the number of days from last vaccination date.

We made phone calls to computer-generated random numbers in the mobile number format belonging to the country’s two national mobile phone network service providers. These providers are responsible for all mobile phone lines in the country. Interviews were conducted via telephone, in English or Chichewa, based on the participants’ preferred language. All respondents aged ≥ 18 years were eligible to participate in the survey upon obtaining verbal consent. Respondents who could not communicate in English or Chichewa were excluded from participating in the survey. A questionnaire was developed in English, translated to Chichewa, and back-translated to English to ensure both English and translated versions had the same meaning. It was then digitized with data range and consistency checks to ensure high data quality. Data were captured on Android tablets using Open Data Kit (ODK) software and stored on a secure online server.

### Sample size

Sample size estimates were based on the primary objective of estimating the weekly rates of self-reported influenza-like or COVID-19-like illnesses (ILI/CLI). Without preliminary data on rates of ILI/CLI in Malawi, we conservatively assumed that 50% of the population had symptoms of ILI/CLI. We assessed that 1537 individuals would allow us to estimate ILI/CLI weekly rates with 2.5% precision. We planned to survey at least 2,000 individuals from the general population per week.

### Study variables

For this analysis, the outcome variable was COVID-19 vaccine receipt. Independent variables included sociodemographic characteristics (sex, age, current district of residence–central, northern, southern, and the highest level of education attained–no education, primary, secondary, tertiary), views on the safety of COVID-19 vaccines (safe, not safe), concern about getting COVID-19 disease (very concerned, not concerned), would get the vaccine, reactions to the vaccine, number of sources of COVID-19 vaccination information reported (one, two, three or more), and decision-making (who had the final say- self, spouse/partner, parents) on vaccine receipt.

### Statistical analysis

We used frequencies and proportions to summarize categorical variables, and medians with interquartile ranges (IQR) were used to summarize continuous variables.

Using cross-tabulations and chi-square tests, we explored potential associations between vaccine receipt and various covariates, including participant age, gender, level of education, and region of residence.

We assessed factors associated with COVID-19 vaccine receipt using multivariable logistic regression. Covariates included in the models were the independent variables listed above. We controlled for potential confounding by including these covariates using backward selection.

### Ethical considerations

The Malawi National Health Sciences Research Committee and Advarra Institutional Review Board in the United States of America (USA) approved the study. Verbal informed consent was obtained from all study participants.

## Results

We enrolled 51,577 adults between July 2021 and April 2022. The median age was 34 years (IQR: 26–43), and most participants were male (65.7%). About 64% of the participants had attained secondary or higher education. Most of the participants resided in the Southern (45.5%) and Central regions (41.2%) (Table 1).

**Table 1:** Sociodemographic characteristics of COVID-19 syndromic surveillance study participants, July 2021 to April 2022. N = 51577.

Variable	Frequency n (%)
**Gender**	
Female n (%)	17693 (34.3%)
Male n (%)	33884 (65.7%)
**Age range (years)**	
(Median 34 years IQR 26–43)	
18–24	9498 (18.4%)
25–34	16622 (32.2%)
35–44	13465 (26.1%)
45–54	8049 (15.6%)
55–64	2421 (4.7%)
65+	1522 (3%)
**Level of Education**	
No Education	1296 (2.5%)
Primary	17191 (33.5%)
Secondary	22402 (43.6%)
Tertiary	10449 (20.4%)
Missing	239
**Current Region of Residence**	
Northern	6845 (13.3%)
Central	21255 (41.2%)
Southern	23477 (45.5%)
**Concern about getting COVID-19 disease**	
Very Concerned	39488 (76.6%)
Little Concerned	12043 (23.4%)
Missing	46
**Thoughts on the Safety of Covid-19 Vaccines**	
Very Safe	37173 (72.2%)
Not at all safe	14310 (27.8%)
Missing	94
**Final Say on Vaccine Receipt**	
Self	46140 (89.6%)
Spouse	2879 (5.6%)
Parents/In-laws	2045 (4%)
Children	186 (0.4%)
Someone else	260 (0.5%)
Missing	47

Overall, 40.2% of the respondents had received at least one dose of a COVID-19 vaccine. There was no significant difference in COVID-19 vaccine receipt between females and males (40.4% vs. 40.2%). The proportion reporting vaccine receipt increased with age, with 27.3% of respondents aged 18 to 24 years compared to 58.2% of the respondents aged 65 years or older. About 54.2% of the respondents with tertiary education, compared to 30.5% without education reported receiving at least one dose of the COVID-19 vaccine (Table 2).

**Table 2:** Factors associated with COVID-19 vaccine receipt among COVID-19 syndromic surveillance study participants, logistic regression, adjusted and unadjusted odds ratios, with 95% confidence intervals and p-values.

Variable	Received Vaccine (Row percentage) Total = 20827	Unadjusted ORs (95% CI)	Adjusted ORs (95% CI)	P-value
**Gender**				
Female	7191 (40.4%)	Ref	1	
Male	13636 (40.2%)	0.99 (0.95–1.03)	0.83 (0.80–0.86)	<0.001
**Age range (years)**				
18–24	2627 (27.3%)	Ref	1	
25–34	5837 (35.1%)	1.44 (1.37–1.53)	1.32 (1.24–1.40)	<0.001
35–44	5883 (43.6%)	2.07 (1.95–2.19)	2.00 (1.88–2.13)	<0.001
45–54	4287 (53.9%)	3.04 (2.85–3.24)	3.02 (2.82–3.24)	<0.001
55–64	1306 (53.9%)	3.13 (2.86–3.43)	3.24 (2.93–3.57)	<0.001
65+	887 (58.2%)	3.73 (3.33–4.17)	3.98 (3.52–4.49)	<0.001
**Level of Education**				
No Education	398 (30.5%)	Ref	1	
Primary	6047 (35.0%)	1.23 (1.10–1.40)	1.30 (1.14–1.48)	<0.001
Secondary	8636 (38.4%)	1.43 (1.27–1.62)	1.76 (1.55–2.01)	<0.001
Tertiary	5657 (54.2%)	2.72 (2.40–3.08)	3.37 (2.95–3.86)	<0.001
**Current Region of Residence**				
Northern	3355 (48.8%)	Ref	1	
Central	9302 (43.6%)	0.81 (0.77–0.86)	0.79 (0.74–0.84)	<0.001
Southern	8170 (34.7%)	0.55 (0.53–0.59)	0.55 (0.52–0.58)	<0.001
**Thoughts on the Safety of COVID-19 Vaccines**				
Very Safe	17962 (48.1%)	Ref	1	
Not at all safe	2865 (19.9%)	0.26 (0.25–0.28)	0.27 (0.26–0.28)	<0.001
**Number of reported sources of COVID-19 vaccine information**				
One source	9364 (38.4%)	Ref	1	
Two sources	7820 (41.2%)	1.12 (1.08–1.17)	1.10 (1.05–1.14)	<0.001
Three or more sources	3467 (45.1%)	1.31 (1.25–1.39)	1.20 (1.13–1.27)	<0.001
**Final Say on Vaccine Receipt**				
Self	18505 (39.9%)	Ref	1	
Spouse	1464 (50.7%)	1.55 (1.44–1.68)	1.52 (1.40–1.65)	<0.001
Parents/In-laws	592 (28.5%)	0.60 (0.54–0.66)	0.89 (0.80–0.99)	0.03
Children	92 (48.4%)	1.38 (1.03–1.84)	0.97 (0.71–1.33)	0.87
Someone else	174 (66.7%)	3.04 (2.35–3.94)	3.43 (2.59–4.55)	<0.001

Adjusting for selected covariates, gender, age, education level, region, perceptions of vaccine safety, number of reported sources of vaccine information, and decision maker on getting vaccinated were independently associated with vaccine receipt. Females were more likely to receive the COVID-19 vaccine than males (AOR 0.83, 95% CI 0.80–0.86). Older age was associated with increased odds of vaccine receipt compared to the younger age group 18–24 years: 25–34 years (AOR 1.32, 95% CI 1.24–1.40), 35–44 years (AOR 2.00, 95% CI 1.88–2.13), 45–54 years (AOR 3.02, 95% CI 2.82–3.24), 55–64 years (AOR 3.24, 95% CI 2.93–3.57) and 65 years+ (AOR 3.98, 95% CI 3.52–4.49). Vaccine receipt was significantly associated with educational status, with the odds of vaccination among individuals with primary education 30% higher (AOR 1.30, 95% CI 1.14–1.48), secondary education 76% higher (AOR 1.76, 95% CI 1.55–2.01), and tertiary education over three times higher (AOR 3.37, 95% CI 2.95–3.85), than individuals with no formal education. Respondents who thought COVID-19 vaccines were unsafe were less likely to receive vaccination than those who thought it was very safe (AOR 0.26, 95% CI 0.25–0.28). The likelihood of COVID-19 vaccine receipt increased with the number of reported sources of vaccine information: AORs 1.10 (95% CI 1.05–1.14) and 1.20 (95% CI 1.13–1.27) for respondents who reported two sources and three or more sources, respectively. The odds of vaccination among respondents whose final decision on vaccine receipt came from their spouse were significantly higher than those who made their own decision (AOR 1.52, 95% CI 1.40–1.65). However, those whose final decision to vaccinate came from parents or In-laws had reduced odds of receiving a vaccination (AOR 0.89, 95% CI 0.80–0.99) than those who made their own decision. Compared to individuals residing in the Northern region, residents of the Central and Southern regions had reduced odds of vaccine receipt (AORs 0.79, 95% CI 0.74–0.84) and 0.55 (95% CI 0.52–0.58), respectively) (Table 2).

About 72.6% of participants reported radio, and 52.1% stated health facilities as primary sources of COVID-19 vaccine information. Other self-reported sources of information on COVID-19 vaccination included social media (16.0%), community leaders (10.7%), family members or friends (10.5%), television (4.4%), schools or workplaces (1.3%), public address systems (1.3%), places of worship (1.0%) and print media (1.0%) (Table 3).

**Table 3:** Self-reported sources of COVID-19 vaccine information among COVID-19 syndromic surveillance study participants, July 2021 to April 2022.

Source of COVID-19 Vaccine Information	Frequency n (%)
Radio	37170 (72.6%)
Healthcare Worker/Health Facility	26662 (52.1%)
social media	8194 (16.0%)
Village/community leader	5480 (10.7%)
family member/friends/community member	5388 (10.5%)
Television	2260 (4.4%)
work/school	643 (1.3%)
Public Address system	663 (1.3%)
Place of Worship	485 (1.0%)
Print Media	502 (1.0%)
text messages	86 (0.2%)
Funerals/weddings/gatherings	48 (0.1%)
Non-Governmental Organization	53 (0.1%)

## Discussion

Our finding that 40% of respondents reported receiving at least one dose of the COVID-19 vaccine is substantially higher than the reported vaccination rate of 7.8% as of May 22, 2022 [20]. This is likely because the scope of our study involved adults with mobile phones who may have had better access to COVID-19 vaccination earlier in the program than those less than 18 years old and individuals living in rural areas [24, 25]. Our study’s demographic characteristics distribution was similar to the country’s mobile phone ownership, with more males than females and most respondents aged between 25 and 44 years [26]. However, it differed from the country’s population profile as 51% are female, and about half are under 18 [16]. It is also worth noting that only about 16% reside in urban areas, and approximately two-thirds of the population is literate [16]. Our findings showed that COVID-19 vaccine receipt varied between different sociodemographic groups. Female gender, increasing age, higher education level, and perception of the vaccines as very safe were associated with an increased likelihood of COVID-19 vaccine receipt.

Females had an increased likelihood of receiving COVID-19 vaccines than males, which was comparable to other studies conducted in Africa on vaccine acceptance [21, 27, 28]. This finding was expected as better health-seeking behaviors have been observed among women compared to males, who have demonstrated hesitancy in seeking healthcare [29]. Improving access to COVID-19 vaccines for men, especially in centers outside the hospital setting, would help increase vaccine receipt in this group.

Older age groups were more likely to have been vaccinated than younger age groups. During the COVID-19 vaccination rollout, those aged 60 years and above, along with frontline workers (such as healthcare workers, police, and prison service employees), were initially prioritized for vaccination [19], which could partly explain why older age groups were more likely to receive a vaccination. A study conducted in Nigeria and a global survey found that older age was associated with increased odds of vaccine acceptance [30, 31]. Older people are at a higher risk of mortality due to COVID-19 than younger people. Older people are also more likely to have a chronic illness, which is associated with COVID-19-related morbidity and mortality [32]. We hypothesize that awareness of these risk factors contributed to an increased likelihood of vaccine receipt in these groups. On the other hand, slower uptake of the vaccine in younger people is contrary to findings from an earlier study in Uganda, which demonstrated a willingness to participate in COVID-19 vaccine trials and an increased likelihood of COVID-19 vaccine acceptance in younger age groups compared to older age groups [28]. The low overall vaccine uptake demonstrated in our study may partly be attributed to poor access to COVID-19 vaccines in this group, given the initial prioritization of vaccines for older individuals.

A higher level of education was associated with increased odds of COVID-19 vaccine receipt, which concurs with other studies that demonstrated factors associated with vaccine acceptance [33]. More educated people are more likely to have access to a broader range of sources of information (including printed media and the Internet), and efforts to improve awareness of the importance and benefits of getting vaccinated than those who are less educated are required.

Concern about the safety of the COVID-19 vaccines was associated with reduced vaccine receipt odds, similar to findings from earlier studies on vaccine acceptance. In a survey conducted among healthcare workers in Malawi, some respondents expressed concern that vaccines developed quickly would be unsafe [34].

Our study also showed that family members significantly influence COVID-19 vaccine receipt, similar to other studies on COVID-19 vaccine receipt [35, 36]. Implementing a community-based approach, where those who received the vaccines would be used as lobbyists to encourage other family members to vaccinate, could help improve vaccine coverage in the country.

Radio is the media source with the highest penetration in Malawi [16]. Our study, unsurprisingly, showed that it had the highest COVID-19 vaccine information coverage, similar to other studies in Malawi [37, 38]. Healthcare workers/health facilities had the second highest coverage of COVID-19 vaccine information, which was expected as most people trust health professionals as a source of health information [39]. Even though it was reported by a sixth of the respondents, social media reached more adults in this survey with COVID-19 vaccination information than other sources of COVID-19 vaccination information. These findings suggest a growing penetration of this platform as a source of health information in the country. Social media, however, can be a source of misinformation also [40], so that a more aggressive approach is required when disseminating health information in these outlets to ensure people access accurate information. The likelihood of COVID-19 vaccine receipt increased with the number of reported sources of vaccine information, which further justifies using multiple channels to broadcast messages about COVID-19 vaccines.

Our study showed that the uptake of COVID-19 vaccines among adults living in Malawi was higher than the national estimates. These findings need to be generalized with caution: first, they only include those aged at least 18 years, and secondly, the study covered a subpopulation of Malawian residents with mobile phone ownership, which may not be representative of the general population.

We recognize the limitations of our study. First, there may have been selection bias, with study participation being limited to adults who had access to an active mobile phone line; we could not reach more than half of the study population, as only about 43% of individuals in Malawi own mobile phones, most of whom reside in urban areas [26]. Second, despite its design, our study could not measure trends in vaccination by month, epidemic waves, or other significant events in response to the pandemic, as only 19% of the sample allows us to estimate the vaccination date. Thirdly, as the survey was based on self-report, and we could not verify the validity of the responses, there may have been social desirability bias. However, due to its large sample size, the study population was well-represented across all demographic characteristics.

## Conclusions

This study found that females, younger and less educated adults were less likely to receive COVID-19 vaccines. Concern about the safety of vaccines was also associated with reduced odds of vaccine receipt. These study findings suggest a need to focus on campaigns that change perceptions of COVID-19 vaccines in males, younger populations, and those less educated. Further research is required to examine barriers to vaccination in these subgroups to develop appropriate messages and channels for better dissemination. Key messages on vaccination need to be crafted to be more applicable and easily accessed by those less educated. More information on the safety of vaccines also needs to be disseminated to the general population to help improve the uptake of vaccines.
